# Effect of hypothyroidism and thyroid autoimmunity on the ovarian reserve: A systematic review and meta‐analysis

**DOI:** 10.1002/rmb2.12427

**Published:** 2021-12-07

**Authors:** Yuko Hasegawa, Yoshikazu Kitahara, Satoko Osuka, Yumiko Tsukui, Mio Kobayashi, Akira Iwase

**Affiliations:** ^1^ Department of Obstetrics and Gynecology Gunma University Graduate School of Medicine Maebashi Japan; ^2^ Department of Obstetrics and Gynecology Nagoya University Graduate School of Medicine Nagoya Japan

**Keywords:** adolescent, AMH, hypothyroidism, ovarian reserve, thyroid autoimmunity

## Abstract

**Background:**

Evidence suggests that hypothyroidism and thyroid autoimmunity (TAI) are possibly associated with ovarian dysfunction. This meta‐analysis aimed to investigate whether hypothyroidism and/or TAI affect the ovarian reserve and evaluated using the anti‐Mullerian hormone (AMH).

**Methods:**

PubMed, EMBASE, Web of Science, and Cochrane Controlled Trials Register databases from inception to October 2020 were searched to identify relevant studies. Studies comparing the AMH levels between the control and the affected groups were included in the data synthesis. The primary endpoint in the meta‐analysis was AMH levels compared with the controls.

**Main findings:**

Nine trials were included in the analysis. The AMH levels were significantly lower in the adults with euthyroid TAI (mean difference −0.12, [95% CI: −0.18 to −0.06]). The AMH levels tended to be lower in subclinical hypothyroidism and overt hypothyroidism than in the control group, although the differences were not significant. The AMH levels were significantly higher in the euthyroid TAI group in the adolescents (mean difference 2.51, [95% CI 1.82 to 3.21]).

**Conclusion:**

TAI and hypothyroidism may affect the ovarian reserve. The opposite effects on AMH levels depending on age suggest that TAI may be implicated in the depletion of follicles in adults following extensive activation of primordial follicles in adolescence.

## 1 INTRODUCTION

Hypothyroidism is a disease that is prevalent in women, even in those of reproductive age.^1^ Thyroid hormones are involved in the control of the menstrual cycle. Oocytes express cell surface receptors for thyroid hormones that affect the actions of follicle‐stimulating hormone and luteinizing hormone through steroid biosynthesis.^2^ As such, thyroid dysfunction disturbs menstrual regularity and ovulation.^3^ In addition, thyroid antibodies are associated with infertility and miscarriage in early pregnancy,^3^ although the mechanism has not been elucidated in detail.

Recently, subclinical hypothyroidism (SCH), defined as an elevated serum thyroid‐stimulating hormone (TSH) level with a normal serum thyroxine level, has been shown to have the same risks of infertility and miscarriage in early pregnancy as overt hypothyroidism (OH).^4^ The prevalence of SCH and OH in women of reproductive age is 4%–8% and 0.3%–0.4%, respectively.^5^, ^6^ Since pregnancy outcomes have been improved by the administration of levothyroxine (LT4) in patients with SCH,^7^ LT4 has been prescribed in clinical practice to keep the serum TSH levels below 2.5 mg/dL.^8^

In addition to infertility and miscarriage, previous studies observed that 20% of patients with premature ovarian insufficiency (POI), defined as a gonadal failure before the age of 40 years based on clinical and laboratory findings, tend to suffer from thyroid autoimmune disorders.^9^ The relationship between POI and thyroid autoimmunity (TAI) is not conclusive. However, thyroid hormone deficiency, elevated TSH levels, and autoimmune mechanisms caused by thyroid autoantibodies may be implicated in POI. This could pose a problem for women desirous of having a baby because even assisted reproductive techniques cannot overcome the depletion of follicles.

Anti‐Mullerian hormone (AMH) is secreted from growing granulosa cells of pre‐antral and early antral follicles and plays a significant role in the regulation of the development of the follicle.^10^, ^11^ The serum concentration of AMH declines with age.^12^ AMH has been established as a reliable marker for the quantitative evaluation of ovarian reserve.^13^ AMH concentrations were shown to be significantly correlated with the oocyte count after ovarian stimulation.^14^ Moreover, numerous studies on AMH, ovarian surgery,^15^, ^16^ and prediction of POI^17^ have been reported. Some studies have implicated the role of SCH or OH^18^, ^19^, ^20^ in ovarian dysfunction, although these investigations have not reached a conclusion because of the variability in the patient background or the cutoff values of TSH in each study.

In the current study, we conducted a systematic meta‐analysis to understand the influence of TAI on ovarian reserve and hypothyroidism.

## 2 MATERIALS AND METHODS

### 2.1 Literature search

A systematic literature review of the PubMed, EMBASE, Web of Science, and Cochrane Controlled Trials Register databases was performed to identify all relevant published studies up to October 2020. The search was limited to human studies published in English, and the following search terms were applied: (ovarian reserve AND hypothyroidism) OR (ovarian reserve AND thyroid autoimmunity) OR (AMH AND hypothyroidism) OR (AMH AND thyroid autoimmunity) OR (ovarian reserve AND thyroiditis) OR (AMH AND thyroiditis). Furthermore, the reference lists of the relevant publications were manually searched for related studies. Two researchers independently completed the literature search and identified the eligible studies.

### 2.2 Study selection

Studies were included whether they satisfied the following criteria: (1) ovarian reserve was evaluated using serum AMH levels; (2) women were diagnosed with SCH, OH, and/or TAI. All prospective, retrospective, and cross‐sectional studies were included. Meanwhile, studies were excluded for the following reasons: (1) publication as an abstract, case report, or review; and (2) failure to provide sufficient data.

### 2.3 Data extraction

Two reviewers independently extracted the following types of data from the included articles: first author, year of publication, patient characteristics, laboratory data including serum levels of TSH, anti‐thyroid peroxidase antibody (TPO‐Ab), anti‐thyroglobulin antibody (TG‐Ab), and AMH. The author was contacted by the corresponding author.

### 2.4 Comparison

The primary analyses aimed to compare the AMH levels between the controls and the women with TAI. Patients with TAI were analyzed separately as adults and adolescents. The secondary analyses compared the AMH levels in the SCH and OH groups with the controls. The outcomes are expressed as the mean weighted difference.

### 2.5 Statistical analysis

The data were pooled using RevMan software (Review Manager Version 5.4, Cochrane Collaboration). The mean AMH values, expressed in ng/mL and standard deviation, were extracted from the original articles. The AMH values reported in pmol/L were converted to ng/mL by dividing these by 7.14. The weighted mean differences were calculated for the AMH values. Heterogeneity between studies was based on the results of the *I^2^* statistics. In the meta‐analysis, a random‐effects model was applied. *p* < .05 was considered statistically significant.

## 3 RESULTS

### 3.1 Literature search

**FIGURE 1:**
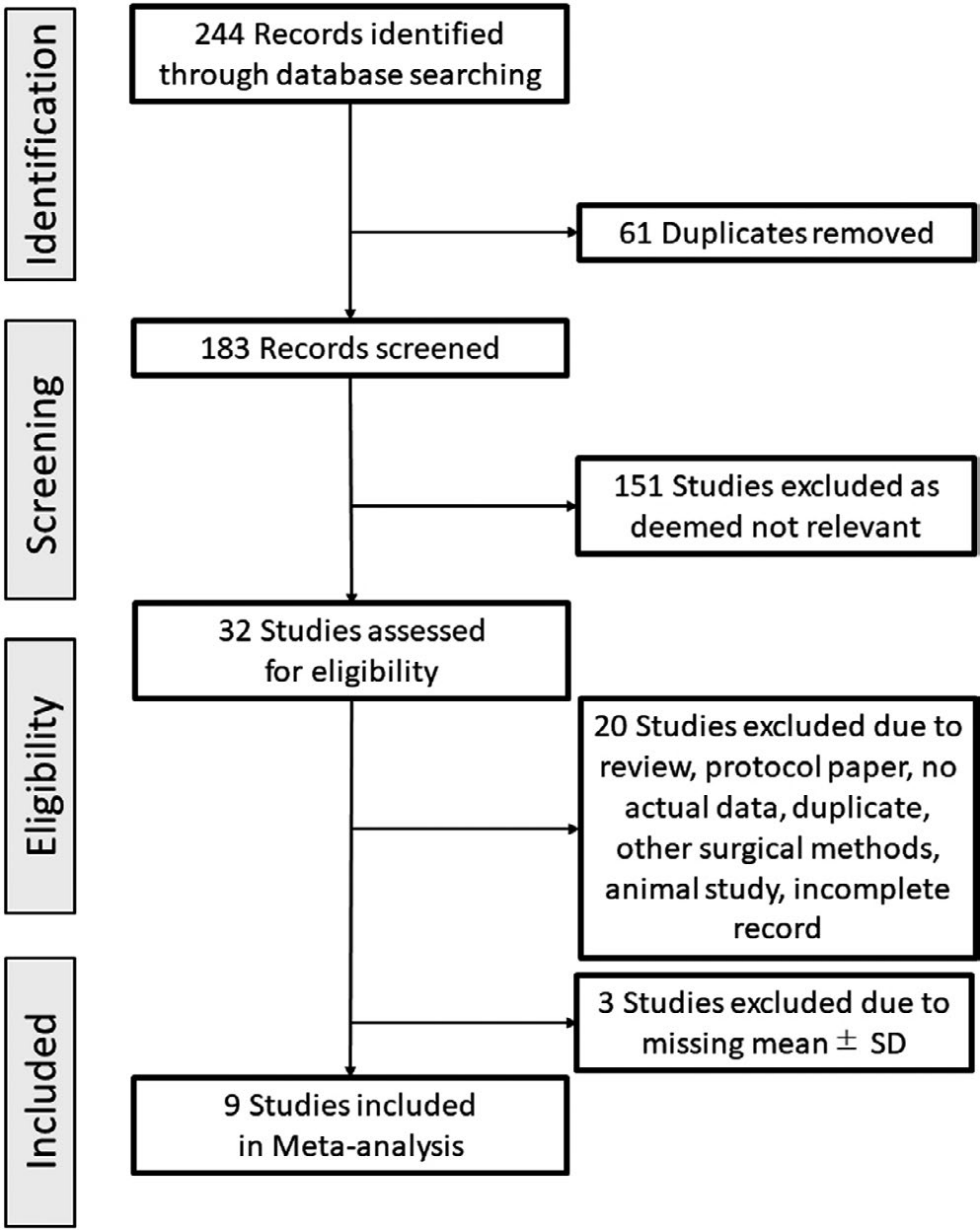
Flow chart for the selection of eligible studies

The literature search in the mentioned databases yielded 244 articles. After removing the duplicate and irrelevant studies, 32 full‐text articles were screened and assessed for eligibility. Thereafter, another 20 articles were excluded for the following reasons: review, protocol paper, no actual data, duplicate, other surgical methods, animal studies, and incomplete records. Furthermore, three studies were excluded due to missing values of the mean AMH levels. Finally, nine full‐text studies were included in the meta‐analysis, as shown in Figure 1.

### 3.2 Characteristics of the included studies

Table 1 provides an overview of the studies comparing TAI and control included in the systematic review. There were two retrospective studies, four case‐control studies, and one cross‐sectional study. Two of the seven studies comparing TAI and control recruited adolescent females,^21^, ^22^ while five studies recruited adults.^20^, ^23^, ^24^, ^25^, ^26^ The inclusion criteria of TSH were 2.5 μIU/ml,^22^ 4.2 μIU/ml,^23^ 5.0 μIU/ml,^26^ and 5.8 μIU/ml,^21^ while three studies did not specify their inclusion criteria for TSH.^20^, ^24^, ^25^ Four studies defined TAI with positivity for TPO‐Ab and/or TG‐Ab,^20^, ^21^, ^23^, ^25^ while two studies defined it only with TPO‐Ab.^22^, ^26^ One study did not specify the inclusion criteria for TAI.^24^

**TABLE 1:** Overview of the studies comparing TAI and control in the systematic review

Study design						Cases			Control		
Author, Year	Design	Thyroid status	Comparison	Inclusion criteria Age (year)	Inclusion criteria TSH (μIU/ml)	*N*	Age (year) Mean ( ± SD)	TSH (μIU/ml) Mean ( ± SD) or [median, (range)]	*N*	Age (year) Mean ( ± SD)	TSH (μIU/ml) Mean ( ± SD) or [median, (range)]
Saglam, 2015	CC	E	TAI vs. control	< 40	NS	85	35.0 ± 2.9	2.7 ± 1.1	82	35.4 ± 2.7	2.1 ± 1.2
Erol, 2016	CC	E	TAI vs. control	12–18	0.36–5.8	57	15.4 ± 1.4	[2.2, (0.6–4.6)]	50	15.1 ± 1.6	[1.97, (0.45–4.6)]
Pirgon, 2016	CC	E	TAI vs. control	Adolescent	< 2.5	30	15.1 ± 1.4	2.5 ± 2.4	30	15.2 ± 1.4	1.8 ± 2.8
Sakar, 2016	CC	E	TAI vs. control	NS	NS	31	30.7 ± 3.8	2.35 ± 1.84	121	30.0 ± 3.4	1.97 ± 2.07
Osuka, 2017	R	E	TAI vs. control	<40	NS	27	34.3 ± 3.9	1.83 ± 1.15	126	34.4 ± 3.9	1.54 ± 0.80
Unuane, 2017	R	E	TAI vs. control	NS	0.01–5.0	187	33.8 ± 4.6	2.02 ± 0.96	2956	32.3 ± 5.0	1.65 ± 0.78
Ke, 2020	CS	E	TAI vs. control	20–40	0.27–4.2	981	31.0 ± 4.4	2.36 ± 0.94	4710	30.6 ± 4.3	2.30 ± 0.90

Abbreviations: CC, case‐control; CS, cross‐sectional; E, euthyroid; NS, not specified; R, retrospective; TAI, thyroid autoimmunity.

Two studies compared the SCH/OH and control groups (Table 2).^19^, ^27^ The cutoff index of TSH for SCH was 3.0 μIU/ml^27^ and 4.2 μIU/ml.^19^ In a study comparing the OH and control groups, the cutoff index of TSH for OH was 10 μIU/ml.^19^ Age is an important since serum levels of AMH decrease with age. The studies included in the review showed comparable mean ages between the case and control groups in each study.

**TABLE 2:** Overview of the studies comparing SCH/OH and control in the systematic review

Study design						Cases			Control	
Author, Year	Design	Comparison	Inclusion criteria Age (y)	Inclusion criteria TSH (μIU/mL)	*N*	Age (year) Mean ( ± SD)	TSH (μIU/ml) Mean ( ± SD) or [median, (range)]	*N*	Age (year) Mean ( ± SD)	TSH (μIU/ml) Mean ( ± SD) or [median, (range)]
Weghofer, 2016	R	SCH vs. control	NS	< 3.0 in control >= 3.0 in SCH	26	38.9 ± 4.4	3.5 ± 0.5	199	38.3 ± 5.1	1.6 ± 0.6
Kucukler, 2018	CC	OH vs. SCH vs. control	20–40	< 4.2 in control 4.2–10 in SCH >= 10 in OH	21in SCH 21 in OH	34.2 ± 4.7 in SCH 35.4 ± 5.9 in OH	4.5 ± 2.0 in SCH 12.1 ± 3.4 in OH	32	32.0 ± 5.1	2.0 ± 1.1

Abbreviations: CC, case‐control; NS, not specified; OH, overt hypothyroidism; R, retrospective; SCH, subclinical hypothyroidism.

### 3.3 TAI and AMH

**FIGURE 2:**
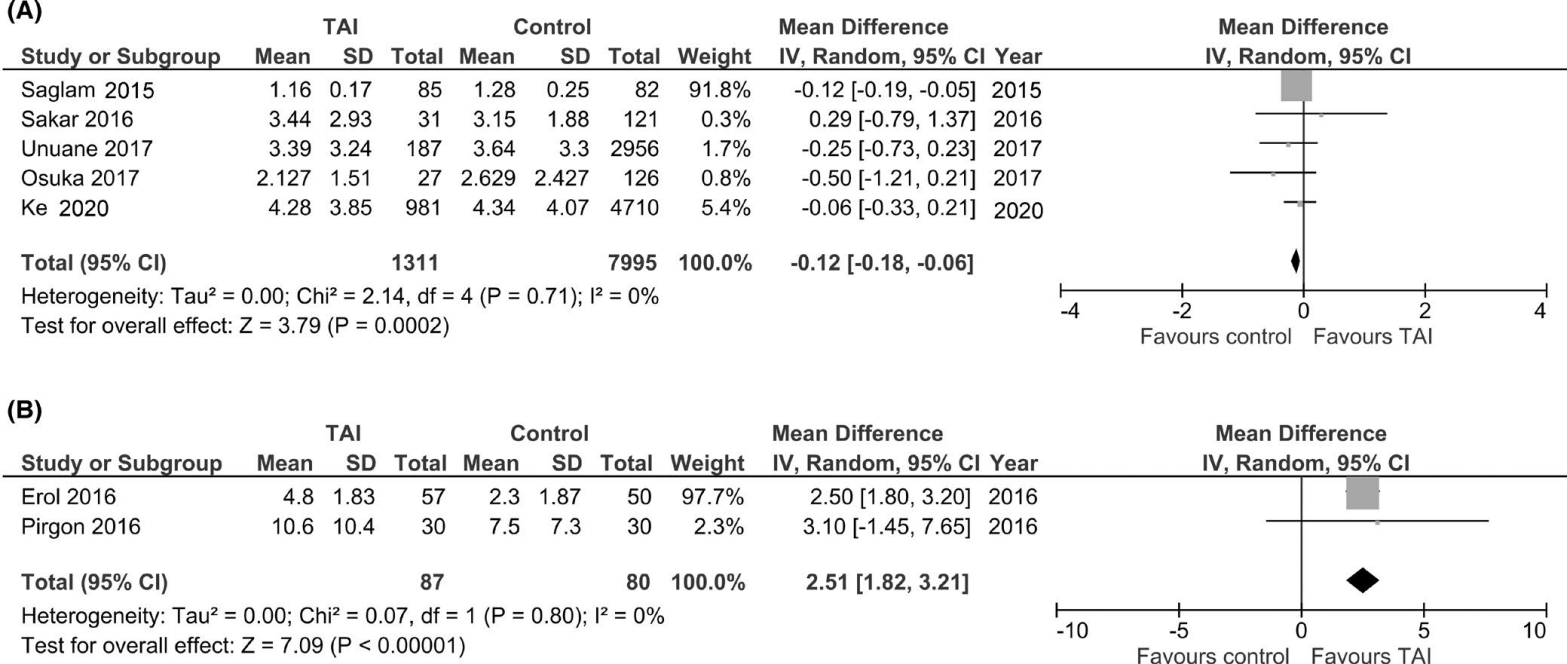
Forest plot (random‐effect model) of the weighted mean differences of the AMH level in women with thyroid autoimmunity (TAI) compared with controls. (A), adults and (B), adolescents

The primary analysis aimed to evaluate the difference in the AMH levels in euthyroid adults with or without thyroid antibodies. The results showed that the AMH levels tended to decline in the group of patients with thyroid antibodies, with a mean difference of −0.12 (95% confidence interval [CI]: −0.18 to −0.06, *I^2^* = 0%; Figure 2A).

We also analyzed the difference in the AMH levels in euthyroid adolescents with and without thyroid antibodies. Interestingly, the results showed that the AMH levels were substantially higher in the group of patients with thyroid antibodies, with a mean difference of 2.51 (95% CI: 1.82 to 3.21; *p* < .00001, *I^2^* = 0%, Figure 2B).

### 3.4 SCH, OH, and AMH

**FIGURE 3:**
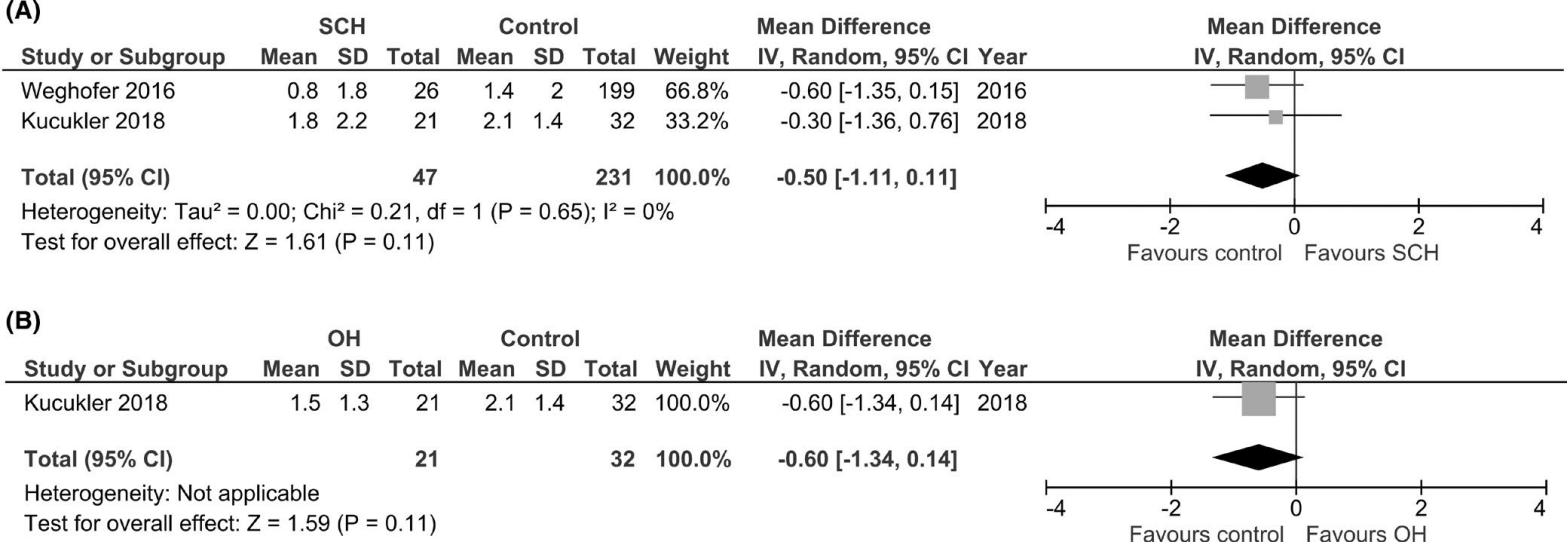
Forest plot (random‐effect model) of the weighted mean differences in the AMH level in women with (A) subclinical hypothyroidism (SCH) and (B) over hypothyroidism (OH) compared with controls

The secondary analysis aimed to evaluate the difference in the AMH levels between a group of patients with SCH and a control group. The results showed that the AMH levels tended to decline in the group of patients with SCH, but there was no significant difference between the groups, with a mean difference of −0.50 (95% CI: −1.11 to 0.11, *I^2^* = 0%, Figure 3A).

We also analyzed the difference in AMH between a group of patients with OH, excluding SCH, and a control group. These data were only reported by Kucukler et al. The results also showed that AMH levels tended to decline in the group of patients with OH, although there was no significant difference between the groups, with a mean difference of −0.60 (95% CI: −1.34 to 0.14, *I^2^* not applicable, Figure 3B).

## 4 DISCUSSION

We conducted a meta‐analysis to examine the effects of three different thyroid dysfunctions on the AMH levels: (1) presence of thyroid autoantibodies in the euthyroid state, (2) SCH, and 3) OH. Compared with the control group, the AMH levels tended to be lower in the adults with SCH and OH, although the differences were not significant. Among patients with thyroid autoantibodies, AMH levels were significantly lower in adults and significantly higher in adolescent girls. The different directions of the effects depending on age may explain why studies on autoimmune hypothyroidism and ovarian reserve have been inconclusive.

The effect of hypothyroidism on the ovarian reserve is thought to involve two main mechanisms: thyroid hormone deficiency and autoantibodies. The study on rats with hypothyroidism induced by propylthiouracil showed that hypothyroidism affected folliculogenesis (more secondary follicles, fewer follicular follicles, and smaller follicle size) and the differentiation of granulosa cells, but it had no effect on the proliferation.^28^ Similar results were reported in a rabbit model.^29^, ^30^ On the contrary, the percentage of atretic follicles in the ovary varied from study to study, remaining the same or increasing.^28^, ^30^

Thyroid hormone inhibits the apoptosis signaling pathway of BAX and caspase‐3 while maintaining the PI3K/AKT pathway active in granulosa cells.^31^ Therefore, thyroid hormone deficiency presumably increased the number of atretic follicles due to the progressive apoptosis of follicles. Since the expression of thyroid hormone transporters and receptors has been reported in human granulosa cells,^32^ the effects of propylthiouracil treatment may be the result of direct action on the ovary by the reduced thyroid hormone levels rather than the elevated TSH levels.^33^ Taken together, a decrease in thyroid hormones causes impairment of folliculogenesis, which prevents the differentiation of granulosa cells and promotes apoptosis into atresia follicles, resulting in lower AMH levels.

Thyroid autoantibodies were measurable in all follicular fluid samples taken from women with thyroid autoimmunity but were completely absent in women without thyroid autoimmunity.^32^ There is a strong correlation between the serum antibody levels and follicular fluid antibody levels,^34^ which shows that plasma TPO‐Ab can move into the follicular fluid. Currently, there have been no experimental studies on the potential pathophysiological mechanisms of thyroid autoantibodies in follicular fluid.

In the clinical setting, a previous study reported that 50% of women with idiopathic POI have anti‐thyroid antibodies and 20% have anti‐ovarian antibodies.^35^ In addition, the fertilization rate and the number of excellent embryos by grading were lower in women with positive thyroid autoantibodies.^34^ Therefore, anti‐thyroid antibodies may be associated with ovarian dysfunction. All things considered the possible decrease in the AMH levels in adult patients with TAI and/or hypothyroidism may have involved both thyroid hormone deficiency and autoantibodies.

Interestingly, stratified analyses by age yielded conflicting results on the AMH levels between adults and adolescents with thyroid autoantibodies. In a cross‐sectional study, the AMH level began to rise at the age of 2 years, declined between 8 and 12 years, and then rose to reach its peak in the mid‐20s.^36^ The increase in the AMH levels at around 2 years old coincides with the onset of growth from the follicle pool. Once the AMH levels have increased, AMH acts to limit the activation of follicle growth, slowing down the recruitment of primordial follicles.^11^, ^37^ This then initiates the decrease in the AMH levels at the age of 8–12 years.

Antibody‐mediated mechanisms lead to the overproduction of free radicals.^38^ Increases in oxidative stress markers and impairment of antioxidant systems have been observed in women with SCH and OH.^39^ In addition, the antioxidant capacities of the serum and follicular fluids are positively correlated.^40^ Primordial, primary, and small antral follicles have been found to exhibit greater resistance to free radicals than other developmental stage follicles.^41^

The increase in serum AMH levels in adolescent females with TAI shows the upregulation of AMH‐producing follicles in a growing follicle pool resulting from the activation of dormant primordial follicles. This may be a compensatory reaction against autoimmune‐mediated damage to growing follicles in a relatively short‐term reaction. The extensive recruitment of primordial follicles following the atresia of damaged growing follicles has been demonstrated in cyclophosphamide‐induced ovarian failure.^42^ This is called the “burn‐out” theory because continuous activation of primordial follicles finally leads to follicle depletion. The current meta‐analysis suggests that a slow “burn‐out” could be involved in the follicle loss caused by TAI.

AMH regulates the selection and growth of the ovarian follicles AMH has been shown to suppress the recruitment of primordial follicles and FSH‐dependent growth.^10^, ^11^ This function of AMH is involved in the prevention of excessive growth of the follicle. In addition, the production of AMH ranges between the subsequent phase of primordial follicle recruitment and initiation of FSH‐dependent growth.^43^ The increase in the pool of growing follicles subsequently results in the possible depletion of follicles and a decrease in the AMH levels. Therefore, we hypothesized the biphasic effects of TAI on the ovarian reserve. In a recent database study, women with Hashimoto's disease aged between 20 and 40 years exhibited a 2.4‐fold higher risk of ovarian failure than controls.^18^ Taken together, the shrinkage of the follicle cohort, indicated by a decline in serum AMH levels, may occur in adult women with TAI.

In light of follicle development in women with TAI and/or hypothyroidism, supplementation with levothyroxine could be an important therapeutic point to recover or maintain ovarian reserve. Kuroda et al. reported that serum AMH levels increased after levothyroxine treatment in women with Hashimoto's disease, but not in the entire SCH group.^44^ This suggests that TAI is more involved in decreasing the AMH levels.

We did not find statistical significance in the decline of AMH, although the AMH levels tended to be lower with SCH and OH. A possible reason behind this is that the age‐dependent decline in AMH disguises the influence of TAI and/or hypothyroidism on ovarian reserve. Other limitations of the study include the cutoff index of TSH and the restricted evaluation of ovarian reserve. SCH was defined using various cutoff indices for TSH. In fact, the inclusion criteria for SCH were different between the studies by Kucukler et al. and Weghofer et al. The American Thyroid Association recommends LT4 supplementation in women seeking assisted reproduction and whose TSH levels are lower than 2.5 μIU/ml.^45^ Therefore, the inclusion criteria of SCH vary from 2.5 μIU/ml to 4.0–5.0 μIU/ml, which is the upper limit of the reference range. This may create difficulty in drawing a firm conclusion on the influence of SCH. Antral follicle count (AFC) is another useful ovarian reserve marker. Few studies have evaluated AFC in women with TAI and/or hypothyroidism than in those with AMH. In addition, the results have been conflicting.^19^, ^46^ Therefore, we performed a meta‐analysis using only AMH.

In conclusion, the current study indicates that TAI and/or hypothyroidism may affect the ovarian reserve. The opposite effects on AMH levels depending on the age suggest that TAI may be implicated in the depletion of follicles in adults following extensive activation of primordial follicles in adolescence. The results also suggest that early intervention in adolescent thyroid diseases may be helpful in maintaining the ovarian reserve. Future studies should pay more attention to the target age group and should be conducted longitudinally, covering the period of adolescence and adulthood. Moreover, both clinical and basic approaches would be helpful in revealing the profiles of follicle growth in each age group.

## CONFLICT OF INTEREST

Yuko Hasegawa, Yoshikazu Kitahara, Satoko Osuka, Yumiko Tsukui, Mio Kobayashi, and Akira Iwase declare no conflict of interest.

## HUMAN/ANIMAL RIGHTS

This review article included no patients, and thus, it did not require approval from Ethics Committee.

## HUMAN RIGHT STATEMENT AND INFORMED CONSENT

This study did not contain any human materials.
